# A Retrospective Observational Study of Rock-Climbing Injuries in Singapore

**DOI:** 10.7759/cureus.71682

**Published:** 2024-10-17

**Authors:** Hui-Shan Angela Lim, Wen Loong Paul Yuen, Raj Socklingam, Ing How Moo, Charles Kon Kam King

**Affiliations:** 1 Orthopedic Surgery, Changi General Hospital, Singapore, SGP; 2 Orthopedics and Traumatology, Changi General Hospital, Singapore, SGP; 3 Orthopedics, Changi General Hospital, Singapore, SGP

**Keywords:** climbing, climbing-related injury, rock climbing, sports injury, trauma

## Abstract

Background

Rock climbing is becoming increasingly popular in Singapore resulting in an increasing number of rock-climbing-associated injuries requiring surgery. The main objective of this study is to evaluate the demographics, mechanism, and extent of injuries in patients admitted following rock-climbing-related injuries over a seven-year period.

Method

All patients admitted to the Department of Orthopedic Surgery between January 2017 and December 2023 with injuries related to rock-climbing accidents were recruited. Data collection was performed retrospectively on the patient profile, day and time of injury, type of rock climbing, mechanism, pattern, and the extent of injury.

Results

Across 22 patients, 68.4% of injuries occurred in the afternoon, distributed equally across the week. Demographically, majority were young adults (27.3 + 7.8 years), female (77.3%) with a mean BMI of 23.67 + 3.27 kg/m^2^. A total of 90.9% of them fell from height commonly resulting in fractures (95.5%), of which 54.5% sustained two or more fractures and 9.5% sustained open fractures. Comparatively, falling from a higher height resulted in multiple injuries, though not statistically significant. The two patients who sustained open fractures fell from a height of 2 m and 10 m. Lower limb injuries (72.7%) were more common than upper limb (18.2%) and spine (22.7%) injuries. A total of 90.9% of them required surgery, of which 36.8% required a second surgery. A relatively equal number of injuries were sustained from bouldering (45.5%) and sport climbing (54.5%). Females were shown to be more likely injured from bouldering than sport climbing (p = 0.040). A total of 100% of bouldering injuries occurred due to falls from height, while 16.7% of injuries during sport climbing were due to laceration or hand-hold injury. Patients who fell from sport climbing fell from a higher height of 3.35 + 2.6 m compared to bouldering at 2.5 + 0.9 m. Moreover, 33.3% of patients injured from sport climbing sustained multiple regions of injuries, two of which were open fractures, while all injured from bouldering only sustained injuries in a single region, only sustained closed fractures, and none affected the upper extremity. A total of 100% of bouldering injures and 80% of sport-climbing injuries required surgery where around 37% of patients in both groups required repeat surgery. Patients with sport-climbing injuries had a longer length of hospital stay (13.8 + 18.3 days) and duration of hospitalization leave (dHL) (81.3 + 45.5 days) compared to bouldering injuries.

Conclusion

Rock-climbing-associated injuries are predominantly caused by falls, commonly resulting in fractures requiring admission and surgical intervention, especially in the lower extremities. With increasing popularity and accessibility of the sport in Singapore, there is an expected increase in the number of orthopedic injuries. Injuries vary widely from lacerations to fractures of the extremities and spine to open fractures requiring emergency surgery. Proper safety precautions, equipment, training, and strict regulations for belay certification should be put in place to mitigate the risk of such injuries.

## Introduction

Rock climbing has reached new heights of popularity in Singapore due to the massive exposure boost since its debut in the 2020 Summer Olympics in Tokyo. There has been a surge in the number of indoor climbing gyms in Singapore with an estimated 40 commercial climbing gyms in 2022, from 13 in 2018 [1].

The sport encompasses multiple disciplines including, but not limited to sport climbing and bouldering [2]. Sport climbing consists of top-rope climbing and lead climbing, with routes typically up to 30 m high. Top-rope climbing involves a person ascending a route with a safety rope attachment from above, while lead climbing involves clipping a rope onto permanent bolts using “quickdraws” that are spaced intermittently from the bottom up, with aims to minimize falls when rock grip is lost. Bouldering is a form of free climbing encompassing a small number of moves that are generally powerful in nature with the use of crash pads to protect climbers from falls. With a wide variety of disciplines in rock climbing, injuries vary widely anatomically from head to toe due to various mechanism of injuries ranging from chronic overuse to acute atraumatic injuries from supraphysiologic loading and to acute traumatic injuries from fall from height or rockfall [3].

Studies on injury patterns have been done in various countries including England, Germany, and the United States of America [4-6]. Jones et al. has reported 50% of climbers sustaining one or more injury in 12 months [5]. With increased accessibility to the sport in Singapore, almost anyone can participate in the sport recreationally with the rental or purchase of a pair of climbing shoes. In turn, an increased number of orthopedic injuries associated with the sport is to be expected [7]. The main objective of this study is to evaluate the demographics, injury pattern, and outcomes of patients admitted with orthopedic injuries as a result of rock climbing in Singapore.

## Materials and methods

This is a retrospective observational study. The waiver of informed consent by the Singhealth Institutional Review Board was granted as only retrospective and de-identified data was used. All patients admitted to the Department of Orthopedic Surgery, in our single orthopedic center, with injuries associated with rock climbing were entered into a registry over a seven-year period between January 2017 and December 2023. This was done by diligently reviewing admissions over the study period and identifying patients who were hospitalized following accidents related to rock climbing. The demographic details of the relevant patients were captured in the registry database. This includes patients who were admitted directly from the Emergency Department and those admitted from the Orthopedic Specialist Outpatient Clinic (SOC). However, this does not include patients with rock-climbing-related injuries admitted under other specialties or those who may have died on the scene, on their way to the hospital, or discharged from the Emergency Department. Patients who were managed solely in SOC were also excluded.

The demographics of the subjects, type of rock climbing, mechanism of injury, type of injuries sustained, and outcomes were traced from the registry database and collated. Demographic variables considered were age, gender, smoking history, and body mass index (BMI). The type of rock climbing was categorized into sport climbing and bouldering. The mechanism of injury includes falls from height and others. Injury patterns include the number and nature of fractures (if any), the number and region of the body affected, and open or closed fractures. The region of the body involved was plotted on the Barell Injury Diagnosis Matrix [8], which is divided into head and neck, spine torso, extremities, and system-wide or unspecified. The extremities are further divided into the upper limb or the lower limb. Outcomes included the requirement for surgical management, length of stay (LOS), duration of hospitalization leave (dHL), and whether an additional surgery was required after discharge.

Descriptive statistics of patient demographic and clinical characteristics were reported as number and percent for categorical data, mean ± standard deviation (SD) for normally distributed data, and median and interquartile range (IQR) for nonnormally distributed data. The Mann-Whitney U-test was used to determine if there was a difference in fall height between patients with multiple vs. single injury, and open vs. no open fractures. Fisher’s exact test was used to determine if there was an association between activity type and (a) sex, (b) multiple injuries, and (c) upper limb injury. Statistical tests were two-sided with a 0.05 significance level. All analyses were conducted using Stata 18 ( StataCorp LLC, College Station, TX).

## Results

Distribution of injuries by day of week and time of day

**Figure 1:**
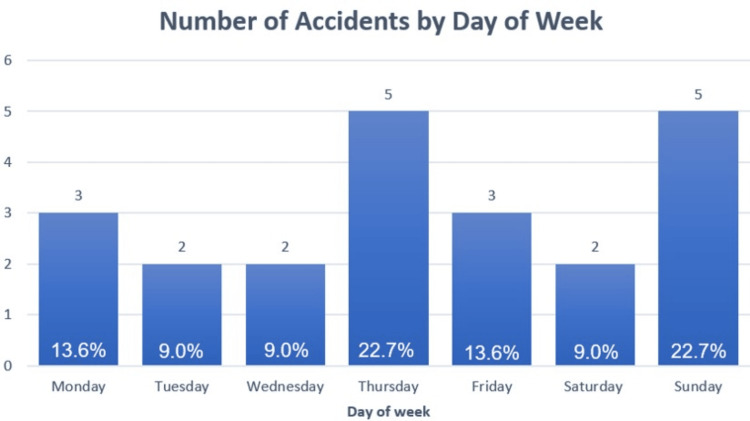
Bar chart distribution of accident occurrence by day of week

A total of 22 patients were included in this study. Rock-climbing-related injuries appear to occur equally throughout the week, with 22.7% (5/22) most commonly occurring on Thursday and similarly on Sunday (Figure 1).

**Figure 2:**
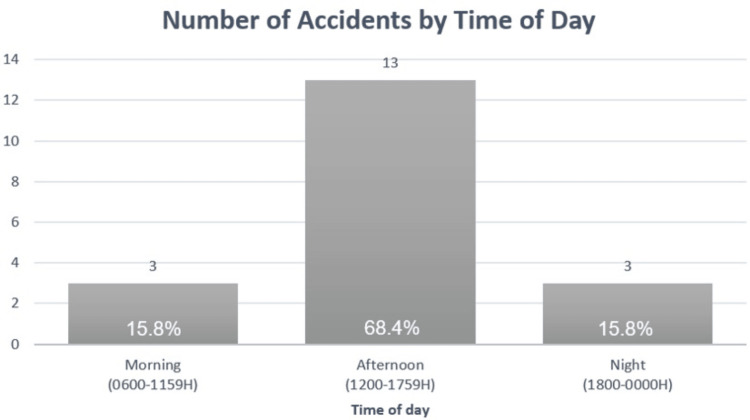
Bar chart distribution of accidents by time of day

For 19 patients with recorded time of injury, majority (68.4%, 13/19) of the accidents occurred in the afternoon (Figure 2). 

General rock climbing: demographics, injury patterns, and outcomes 

All patient demographics, injury patterns, and outcomes of rock-climbing-related orthopedic injuries are summarized in Table 1.

**Table 1:** Summary of demographics, injury patterns, and outcomes of all rock-climbing injuries ^1 ^Excluding the one patient who did not sustain a fracture; *excluding one out of the 22 patients declined surgery due to cost concerns; ^across patients with surgeries on the first admission

Variable		Total (n = 22)
Age (years)		27.3 + 7.6
Gender		
	Female	17 (77.3%)
	Male	5 (22.7%)
Smoker		2 (9.1%)
BMI (kg/m^2^)		23.67 + 3.27
	BMI >23	5 (45.5%)
	BMI <23	6 (54.5%)
Mechanism of injury		
	Fall from height	20 (90.9%)
	Others	2 (9.1%)
Fall height (m)		2.94 + 2.01
No. of region of injuries		
	Single region	18 (81.8%)
	Multiple regions	4 (18.2%)
No. of bones fractured		
	0	1 (4.5%)
	1	9 (40.9%)
	>2	12 (54.5%)
Type of fracture ^1^		
	Open	2 (9.5%)
	Closed	19 (90.5%)
Region of injury		
	Upper limb	4 (18.2%)
	Lower limb	16 (72.7%)
	Spine	5 (22.7%)
No. of operations *		
	0	2 (9.5%)
	1	15 (71.4%)
	>2	4 (19%)
Additional surgery ^		7 (36.8%)
LOS (days)		10.5 + 14.4
dHL (days)		76.3 + 39.1

Rock-climbing injuries occurred mostly in young adults with a mean age of 27.3 + 7.8 years and females (77.3%). The mean BMI was 23.67 + 3.27 kg/m^2^, of which 45.5% were overweight with BMI > 23 kg/m^2^. The most common mechanism of injury was from fall from height (90.9%), with the mean fall height of 2.94 + 2.01 m. A total of 21 of the patients (95.5%) sustained fractures, of which five (23.8%) sustained two or more fractures, and two (9.5%) sustained open fractures. Overall, lower limb injuries (72.7%) were more common than upper limb (18.2%) and spine (22.7%) injuries. Excluding the single patient who declined surgery due to cost concerns, 19 of them (90.9%) required surgery, within which seven (36.8%) required a second surgery related to the initial injury for reasons such as removal of implants, change of implants or additional ligamentous repair. The mean LOS was 10.5 + 14.4 days, and the patients required an average dHL of 76.3 + 39.1 days.

In assessing injury patterns with age, those with multiple injuries had a median age of 24 years compared to 26 years (IQR: 21.8-29.8) with a single injury, though not statistically significant (p = 0.668). The age of the two patients with open fracture was 27 and 39 years, while the median age of patients with closed fractures was 25 years (IQR: 21.0-29.0). In assessing injury patterns with gender, three of four patients (75%) who sustained multiple injuries were females (p = 0.910), and both patients who sustained open fractures were females (p = 0.445). In assessing injury patterns with BMI, the median BMI among patients with multiple injuries was 21.1 kg/m^2^, compared to 24.8 kg/m^2^ (IQR: 20.9-26.7) among patients with a single injury, though not statistically significant (p = 0.463). The BMI of one patient with open fracture was 20.5 kg/m^2^, while the median BMI of patients with closed fractures was 23.2 kg/m^2^ (IQR: 20.9-26.7), though not statistically significant (p = 0.365). In assessing injury patterns with respect to fall height, the median fall height among four patients with multiple injuries was 3 m (IQR: 2.3-6.5), compared to 2 m (IQR: 1.5-3) among 15 patients with a single injury. There was a lack of evidence of a difference in fall height between the two groups (p = 0.352). The fall height among two patients with open fractures were 2 m and 10 m, while the median fall height among 17 patients with no open fractures was 2 m (IQR: 1.5-3). There was no evidence of a difference in fall height between the two groups (p = 0.336).

Sport climbing vs bouldering: demographics, injury patterns, and outcomes 

All patient demographics, injury patterns, and outcomes in relation to the type of rock-climbing injuries are summarized in Table 2.

**Table 2:** Demographics, injury patterns and outcomes of sport climbing vs bouldering injuries ^1^Excluding the one patient who did not sustain a fracture; *excluding one out of the 22 patients as declined surgery due to cost concerns; ^across patients with surgeries on first admission

Variable		Sport climbing	Bouldering	p-value
		12 (45.5%)	10 (54.5%)	
Age (years)		28.7 + 9.0	25.7 + 5.0	0.597
Gender				0.040
	Female	7 (58.3%)	10 (100%)	
	Male	5 (41.7%)	0 (0%)	
Smoker		2 (16.7%)	0 (0%)	0.481
BMI (kg/m^2^)		23.0 + 2.8	24.45 + 3.65	0.715
	BMI > 23	2 (33.3%)	3 (60%)	
	BMI < 23	4 (66.7%)	2 (40%)	
Mechanism of injury				0.481
	Fall from height	10 (83.3%)	10 (100%)	
	Others	2 (16.7%)	0 (0%)	
Fall height (m)		3.35 + 2.6	2.5 + 0.9	0.833
No. of region of injuries				0.096
	Single region	8 (33.3%)	10 (100%)	
	Multiple regions	4 (66.7%)	0 (0%)	
No. of bones fractured				
	0	1 (8.3%)	4 (40%)	0.626
	1	5 (41.7%)	6 (60%)	
	>2	6 (50%)	0 (0%)	
Type of fracture^1^				0.481
	Open	2 (18.2%)	0 (0%)	
	Closed	9 (81.8%)	10 (100%)	
Region of injury				
	Upper limb	4 (33.3%)	10 (100%)	0.096
	Lower limb	8 (66.7%)	8 (80%)	0.646
	Spine	3 (25%)	2 (20%)	1.000
No. of operations*				0.220
	0	0 (0%)	2 (20%)	
	1	8 (72.7%)	7 (70%)	
	>2	3 (27.3%)	1 (10%)	
Additional surgery^		4 (36.4%)	3 (37.5%)	0.663
LOS (days)		13.8 + 18.3	6.7 + 5.4	0.234
dHL (days)		81.3 + 45.5	70.3 + 28.6	0.341

Across the 22 patients, 10 (45.5%) were injured from bouldering and 12 (54.5%) from sport climbing. The mean age of patients injured from sport climbing was 28.7 + 9.0 years, while bouldering was 25.7 + 5.0 years (p = 0.597). A total of 100% (10/10) of patients who were injured from bouldering were females, compared to 58.3% (7/12) of patients who were injured from sport climbing (p = 0.040). Patients injured from bouldering had a higher mean BMI of 24.45 + 3.65 kg/m^2^ with 60% (3/6) of them overweight compared to those injured from sport climbing with a mean BMI of 23.0 + 2.8 kg/m^2^ with 33.3% (2/6) of them overweight (p = 0.715). A total of 100% of bouldering injuries were sustained from falling from height, while sport climbing had 16.7% (2/12) of the patients injured due to other reasons: laceration or hand-hold injury (p = 0.481). The mean fall height in sport climbing was 3.35 + 2.6 m which was higher than that in bouldering 2.5 + 0.9 m (p = 0.833). No patient (0/10) who was injured from bouldering had multiple injuries, compared to 33.3% (4/12) of patients who were injured from sport climbing (p = 0.096). All of the patients injured from bouldering only sustained single fractures, while two of the 21 patients (9.5%) with open fractures were injured from sport climbing compared to bouldering, where all patients only sustained closed fractures (p = 0.481). Similarly, no patient (0/10) who was injured from bouldering had an upper limb injury, compared to 33.3% (4/12) of patients who were injured from sport climbing (p = 0.096). A total of 20% (2/10) of those injured from bouldering did not require surgery, while all injuries from sport climbing required surgery, three of which (27.3%) required two or more operations (p = 0.481). Both injuries from sport climbing and bouldering had a relatively equal need for a repeat surgery of 36.4% (3/11) and 37.5% (3/8), respectively, (p = 1.000). For sport-climbing injuries, the mean LOS is 13.8 + 18.3 days and the dHL is 81.3 + 45.5 days which are comparatively higher (p = 0.573) than those in bouldering injuries with a mean LOS of 6.7 + 5.4 days and mean dHL of 70.3 + 28.6 days (p = 0.692).

## Discussion

To our knowledge, this is the first study to assess inpatient orthopedic injuries related to rock climbing in Singapore. With increasing popularity and accessibility to the sport, there is an increase in the number of orthopedic injuries as a result of rock-climbing accidents globally. In indoor bouldering and sport climbing with an auto-belay system, beginners can simply start after a short safety briefing and the rental of a climbing shoe. Instructor-led lessons are available, but not compulsory. As a result, beginners who have yet to develop the proper technique and muscle strength for specific movements including falling techniques are significantly more prone to accidents with injuries. In identifying potential risk factors and injury patterns of these injuries, further caution and preventive measures can be put in place.

Based on our study, rock-climbing-associated injuries requiring hospitalization tend to occur more commonly over the weekend afternoons, likely during their free time outside of working hours. Due to long work hours during the weekdays, many people compress their weekly exercise on the weekend, giving rise to the colloquial term “weekend warrior” [9]. Robert et al. reported that those who participated in physical activity on the weekend had a higher rate of severe injury than those who exercised on weekdays [10]. Therefore, there needs to be further emphasis on safety procedures to prevent rock-climbing-associated injuries.

Current literature on assessing demographics such as age, gender, and BMI as significant risk factors for injury is conflicting [11]. Our study suggests that while age and BMI are not a predictive factor of injury, females are significantly more likely to sustain fractures requiring surgery from acute rock-climbing injuries, especially in bouldering compared to sport climbing. Women athletes are reported to be have 40% to 75% muscle strength compared to men [12], with the difference in absolute strength between the genders more evident in the upper body than the lower body [13]. Few studies have suggested that muscular strength and grip strength are potential risk factors to musculoskeletal injuries [14]. Therefore, this could explain why females may be at higher risk of injury, especially in bouldering which requires burst power that may be lacking in females. However, this finding is in contrast to a study by Nelson et al. that reported females having a higher risk of sprain and strains while males were more prone to lacerations and fractures [15]. Overall, it may be difficult to draw this conclusion as other factors such as years of climbing experience, skill level [16,17], and level of difficulty of the climbs based on the standardized International Union of Alpine Associations (UIAA) scale [18], which were shown as potential risk factors, were not assessed in this study.

Climbing injuries are commonly due to traumatic or overuse injuries, mostly affecting the extremities. Overuse injuries tend to be chronic [19] due to overstrain from strenuous repetitive movements and are commonly followed up in the outpatient setting. They are associated with a higher incidence of upper extremity injuries [20], especially in sport climbing. On the other hand, traumatic injuries occur acutely, commonly due to impacts from a fall or wall collision [3]. They tend to involve the lower extremities or trunk in comparison to overuse injuries [20,21], are more prevalent in bouldering [7], and commonly result in fractures that require hospitalization [21]. Lower limb injuries include sprains, dislocations, ligament or quadricep injuries, or ankle fractures [22-24]. This corresponds with our study which reports hospitalized patients of which majority sustained lower extremity injuries, predominantly fractures, due to fall from height and requiring surgery. Spine injuries account for up to 20% of fractures [16] which coincides with our study where 22.7% of the patients sustained spinal fractures. Hence, with increased fracture risks associated with rock climbing, more hospital resources are required as the sport continues to gain popularity.

In sport climbing, there are two forms of belays available: traditional manual belay system or auto belay system. In Singapore, the Singapore National Climbing Standards (SNCS) was introduced in 1998 which consists of a series of certification courses for traditional manual belaying that are endorsed by the Singapore Sports Council and administered and regulated by the Singapore Sports Climbing and Mountaineering Federation (SSCMF) [25]. Despite regulations put in place, not all local climbing gyms require SNCS certification and instead simply require climbers to pass competency tests (also known as “verification tests”) developed by the gyms themselves [26] with the introduction of assisted braking devices (ABD). ABD is an auto belay system that acts to hold a falling/hanging climber and is believed to help minimize accidents where the belayer is unable to catch the climber due to burns or inability to catch the full force of the fall [27]. Interestingly, despite the protection of a rope via an auto or manual belayer in sport climbing, our study reports 83.3% of injuries due to fall from height, though without specifics on the underlying causes. While Daniel et al. reports that most belay-related climbing incidents point to inadequate attentiveness and technical skills [28], further studies are needed on the underlying causes such as human error vs mechanical belay failure. Overall, there should be increased emphasis on safety education with stricter regulations on safety belay certification to reduce the risk of rock-climbing-associated injuries.

There are several limitations to this study. Firstly, this study comprises of a small population of 23 patients requiring admission which may not be representative as it lacks cases that were followed up outpatient. Therefore, further research should be done with a larger population size and extended study duration to improve the reliability and representation of the cohort study. Secondly, this study has a retrospective design where the data collected relied on the accuracy and completeness of the registry and electronic medical records. Due to the incompleteness of the data registry, there is a lack of study on potential risk factors such as indoor vs outdoor climbing, skill level, climbing difficulty, and differentiation between top-rope climbing and lead climbing.

## Conclusions

Rock-climbing-associated injuries are predominantly caused by falls, often leading to fractures that require hospitalization and surgical intervention, particularly in the lower extremities. In bouldering, compared to sport climbing, there is a higher prevalence of females sustaining injuries due to the lower muscular and grip strength compared to males. As the sport continues to gain popularity and become more accessible in Singapore, a corresponding increase in the number of orthopedic injuries is expected. These injuries can range from minor lacerations to more severe conditions like extremity fractures, spinal injuries, and open fractures that require emergency surgical treatment.

In light of these risks, it is crucial to emphasize the importance of preventive measures, catering them to the different genders and types of rock climbing. Comprehensive safety protocols, including the proper use of climbing equipment, rigorous training programs, and strict adherence to safety standards, play a vital role in reducing the incidence of injuries. Additionally, implementing mandatory belay certification and regular inspections of climbing facilities can further ensure the safety of participants. With a collective effort from climbers, facility managers, and healthcare professionals, many of the severe injuries associated with this activity can be effectively mitigated, ensuring that rock climbing remains a safe and enjoyable sport for all.
